# Vascular Resection for Pancreatic Cancer: 2019 French Recommendations Based on a Literature Review From 2008 to 6-2019

**DOI:** 10.3389/fonc.2020.00040

**Published:** 2020-02-04

**Authors:** Jean Robert Delpero, Alain Sauvanet

**Affiliations:** ^1^Institut Paoli-Calmettes (IPC), Marseille, France; ^2^Faculté de Médecine, Aix Marseille Université, Marseille, France; ^3^Hôpital Beaujon, Clichy, France; ^4^Université Paris VII - Denis Diderot, Paris, France

**Keywords:** pancreatic adenocarcinoma, recommendations (guidelines), French recommendations, venous resection, arterial resection

## Abstract

**Introduction:** Vascular resection remains a subject of debate in the management of Pancreatic Ductal Adenocarcinoma (PDAC). These French recommendations were drafted on behalf of the French National Institute of Cancer (INCA-2019).

**Material and Methods:** A systematic literature search, with PubMed, *Medline*® (OvidSP), EMBASE, the Cochrane Library, was performed for abstracts published in English from January 2008 to June 2019, and identified systematic reviews/metaanalyses, retrospective analyses and case series dedicated to vascular resections in the setting of PDAC. All selected articles were graded for level of evidence and strength of recommendation was given according to the GRADE system.

**Results:** Neoadjuvant treatment should be performed rather than direct surgery in borderline and locally advanced non-metastatic PDAC with venous and/or arterial infiltration (T4 stage). Patients who respond or those with stable disease and good performance status should undergo surgical exploration to assess resectability because cross-sectional imaging often fails to identify the extent of the remaining viable tumor. Combining vascular resection with pancreatectomy in these cases increases the feasibility of curative resection which is still the only option to improve long-term survival. Venous resection (VR) is recommended if resection is possible in the presence of limited lateral or circumferential involvement but without venous occlusion and in the absence of arterial contact with the celiac axis (CA; cephalic tumors) or the superior mesenteric artery (SMA; all tumor locations) (Grade B). The patients should be in good general condition because mortality and morbidity are higher than following pancreatectomy without VR (Grade B). In case of planned VR, neoadjuvant treatment is recommended since it improves both rate of R0 resections and survival compared to upfront surgery (Grade B). Due to their complexity and specificities, arterial resection (AR; mainly the hepatic artery (HA) or the CA) must be discussed in selected patients, in multidisciplinary team meetings in tertiary referral centers, according to the tumor location and the type of arterial extension. In case of invasion of a short segment of the common HA, resection with arterial reconstruction may be proposed after neoadjuvant therapy. In case of SMA invasion, neoadjuvant therapy may be followed by laparotomy with dissection and biopsy of peri-arterial tissues. A pancreaticoduodenectomy (PD) with SMA-resection is not recommended if the frozen section examination is positive (Grade C). In case of distal PDAC with invasion of the CA, a distal pancreatectomy with CA-resection without arterial reconstruction may be proposed after neoadjuvant therapy and radiologic embolization of the CA branches (expert opinion).

**Conclusion:** For PDAC with vascular involvement, neoadjuvant treatment followed by pancreatectomy with venous resection or even arterial resection can be proposed as a curative option in selected patients with selected vascular involvement.

## Introduction—Resectability of Pancreatic Ductal Adenocarcinomas (PDAC)

Selection of patients for vascular resection is based on the probability of obtaining complete surgical resection (R0), because unlike R1 resection, this can result in prolonged survival or even be curative [Level of Evidence (LE) 3] (1–7). The presence and extent of vascular involvement are determined on high-quality thin-section images, with an anatomical basis for the classification of tumors as “borderline resectable” or “locally advanced” but not metastatic (8, 9). Many classifications have been used to define the extent of PDAC, which is based on the relationship between the tumor and the venous or arterial axes (10, 11) (LE 3). The most common system is the National Comprehensive Cancer Network's (NCCN) classification, updated in November 2018 (12) (LE 2) (Supplemental Material 1). The notion of a “borderline” tumor has recently changed to take into account the anatomical classification, the probability of a histologically incomplete resection (R1), the patient's clinical status (general condition, co-morbidities, performance-status, “fragility syndrome”) and the “biological” status of the disease (LE 2) (13–16). The International Consensus on the definition of “borderline” tumors recommends to use a threshold CA 19-9 rate ≥ 500 units/mL for the latter (14) (LE 3) (Supplemental Materials 2, 3).

A recent study (17) has shown that a standardized pathological protocol R0-resection based on 1 mm clearance was rarely achieved after upfront venous resection due to microscopic involvement of the SMV-groove (LE 4). It is important to note that patients considered to be at high risk of R1 resection and/or those with an unfavorable clinical and/or “biological” status are now candidates for neoadjuvant therapy (18–26) (LE 3). In one North American study (27) the benefits of neoadjuvant therapy were found to be significant in the presence of “unilateral” venous involvement (Ishikawa II-III) (LE 3). The PV patency ratio and its improvement under treatment are new prognostic indicators for PDAC treated with preoperative chemo-radiotherapy (28) (LE 4).

For borderline resectable PDAC, several more recent studies including two meta-analyses (29, 30) (LE 3), one phase II trial (31) and one randomized controlled trial (32) (LE 2), have confirmed that survival was improved after neo-adjuvant therapy followed by surgery than after upfront surgery followed by adjuvant therapy, even in an intent-to-treat analysis. The NCCN recommendations version 1.2019 (November 8, 2018) state that: “Immediate” resection of borderline tumors is no longer recommended (unlike 2016 recommendations), despite the absence of a randomized trial (neoadjuvant therapy vs. “immediate” surgery) and the definition of the best therapeutic protocol to use” (12, 15) (LE 2).

The purpose of neoadjuvant therapy is to increase the rate of patients candidates for potentially curative secondary resection. A systematic review published in 2017 (33) compared the pathological data in patients who underwent “upfront” surgery to those who underwent surgery after “neoadjuvant treatment.” A significant reduction in the relative risk (RR) of R1 resection (RR = 0.66) and other negative predictive factors (tumor size, lymph node metastases, perineural extension, and lymphatic emboli) were observed after neoadjuvant treatment (LE 3) (Supplemental Material 4).

Due to the high prevalence of “borderline resectable” and “locally advanced” PDAC (around 15 and 25% respectively) and the lack of consensus about the treatment of theses entities, our aim was to establish recommendations regarding the treatment of PDAC with vascular involvement based on the existing literature.

## Methodology

The National Institute of Cancer (INCa) commissioned these Guidelines in January 2017 and appointed a guideline leader (chair A.S.) who invited selected authors, all involved in the management of PDAC, to participate in the project development (May 2017). The key questions were prepared by the coordinating team and then approved by the other members. The coordinating team formed task-force subgroups, each with its own leader (J.R.D. for surgery), and divided the key topics among these task forces (October 2017). Process and steps taken to reach the final recommendations were illustrated in Table 1.

**Table 1:**
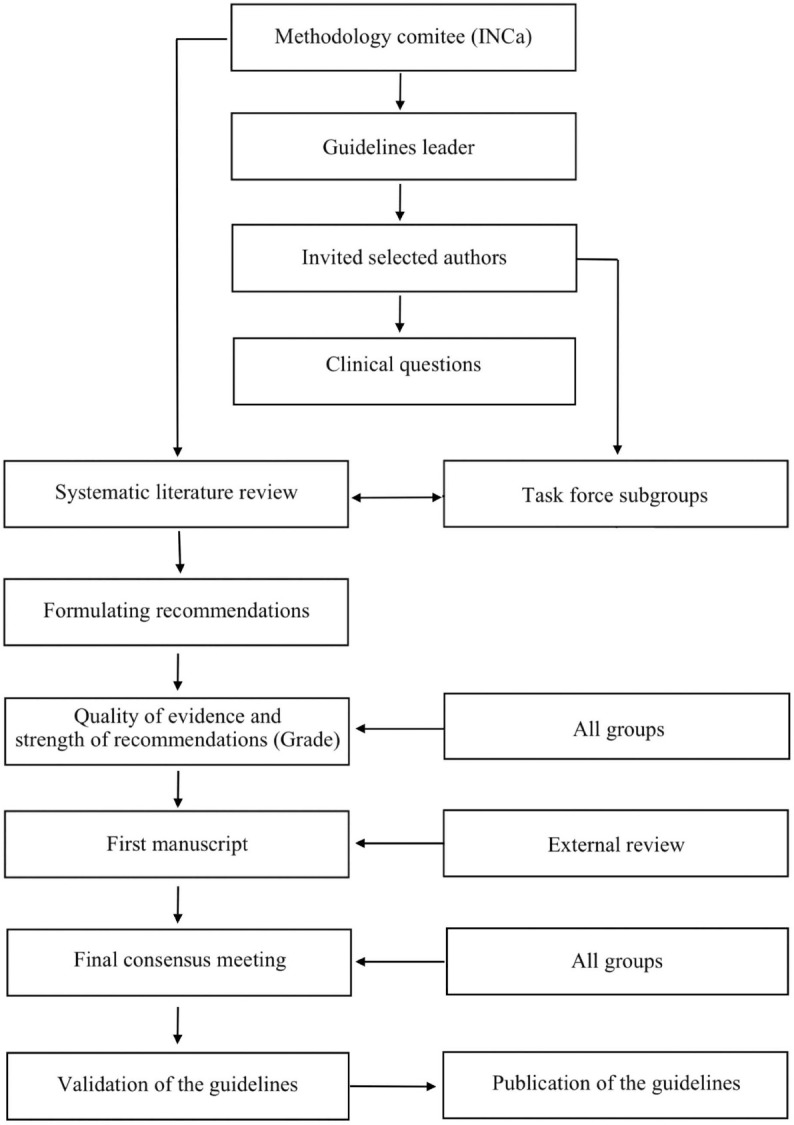
Flow chart of process and steps taken to reach the final recommendations.

The INCa team independently performed systematic literature searches, with PubMed, *Medline*® (OvidSP), EMBASE, the Cochrane Library, and the internet for abstracts published from January 2008 to December 2017. Each task force also performed a systematic literature search. The literature search was restricted to abstracts published in English. Searches were updated every 3 months until June 2019. The search focused on fully published randomized controlled trials (RCTs), meta-analyses, prospective series and national and international guidelines and consensus. However, the literature search concerning vascular resections identified no RCT, only 4 systematic reviews/metaanalyses on venous resection and 2 systematic reviews/meta-analysis on arterial resection; thus, retrospective analyses and case series were also included. Conversely all case reports were excluded.

Manuscripts from abstracts containing relevant data were included. A summary of each reviewed manuscript was completed and summarized in literature tables for each key topic to prepare evidence-based and well-balanced statements on the assigned key questions for each task force. All selected articles were graded by the level of evidence and strength of recommendation according to The Grading of Recommendations Assessment, Development, and Evaluation (GRADE) system (Supplemental Material Methodology).

Each task force developed a draft and proposed statements on their assigned key questions, which were discussed on 4 plenary meetings (from November 2017 to September 2018). Recommendations were formulated based on the available evidence. All recommendations included a Grade rating based on the quality of evidence and strength of recommendation (Supplemental Material Methodology).

A first synthesis of the work from different groups was completed in January 2019. Thereafter, a combined document with all recommendations was created, which was reviewed and approved by all the group leaders, finalized and submitted to a national external review by 70 physicians, oncologists and surgeons (out of 126 solicited by the INCa), selected by regional cancer networks and 17 scientific societies collaborating in this project. The final manuscript was drafted after taking into account all comments and answering questions from the external validation group (April 2019) (Supplemental Material Methodology). All authors agreed on the final draft of the manuscript containing the recommendations. After agreement of all group members during a final plenary session held in Paris in May 2019, the guidelines was published online in November 2019 (https://www.e-cancer.fr).

These Guidelines will be considered for review every year or sooner if new and relevant evidence becomes available. An update will be done every 3 years after the publication of the recommendations. Any updates to the Guidelines in the interim will be noted on the INCa website.

These Guidelines are an official statement of the French Association of Hepatobiliary and Pancreatic Surgery and Liver Transplantation (ACHBT). It provides practical advice on how to manage pancreatic adenocarcinoma.

## Vascular Resection for Pancreatic Cancer

### Venous Resection

#### “What Are the Indications of Venous Resection?”

##### Recommendations

Venous resection associated with pancreatectomy is recommended if resection is possible in the presence of limited lateral or circumferential involvement but without venous occlusion and in the absence of arterial contact with the celiac trunk (cephalic tumors) or the superior mesenteric artery (all tumor locations) (Grade B).

The patients should be in good general condition because mortality and morbidity are higher than in pancreatectomy without venous resection (Grade B).

In the case of a planned venous resection, neoadjuvant treatment is recommended since it improves the rate of R0 resections and survival (Grade B).

##### Comments

Performing venous resection (VR) followed by reconstruction of the mesenteric-portal venous axis during pancreatic resections for PDAC may allow “en-bloc” resection facilitated by the superior mesenteric artery (SMA) “first” approach (34, 35). Pancreaticoduodenectomies (PDs) are associated with VR in up to 25% of cases in France and Europe (less frequently in the US and more frequently in Japan). Distal pancreatic resections are associated with VR in 5–35% of cases (36–39) (LE 4), and in 12% of cases according to a survey by the French Association of Surgery (40) (LE 3) (Table 2). Total pancreatectomies (TP) are performed in more than 50% of cases with VR (57% according to a survey by the French Association of Surgery) (41) (LE 4).

1. The decision to perform a VR may be planned or unplanned based on a possible intraoperative diagnosis of extension limited to the venous axis [Superior Mesenteric Vein (SMV); venous confluence, portal vein (PV)]. Unplanned pancreatectomies with venous resections were associated with more R1 resections in a retrospective study (42) (LE 4).
2. The anatomical location and extent of the PDAC has a fundamental impact on the location and length of the VR [PV, venous confluence, SMV or SMV plus one of its associated branches (the first jejunal vein) (43–49) (LE 3)]. If a tumor of the neck or the right part of the pancreatic body invades the venous axis, the PD can be extended to the left, with the pancreatic section located to the left of the median line at the origin of the splenic artery, and the venous resection performed “en bloc” (50) (LE 4). In case of a segmental VR during distal pancreatectomy, the veins draining the cephalic pancreas actually limit mobilization of both venous extremities and increase the need for an interposition graft for reconstruction (39) (LE 4).
3. The length of the VR has a negative prognostic value “*per se*” because it reflects the extent of disease (51, 52). The threshold associated with a poorer prognosis is > 2 cm (41, 51) (LE 3), or > 3 cm (52) (LE 4). In the case of locally advanced cancer and complex VR (venous occlusion with portal hypertension), the temporary use of a mesenteric-portal shunt to limit the duration of hepatic venous ischemia has been suggested (53–55) (LE 4).
4. The reconstruction technique depends on the type and length of the VR (LE 4): The technique may include a lateral resection followed by a direct suture or autologous patch venoplasty [type 1-2 VR according to the ISGPS classification (56)], a segmental resection followed by direct end-to-end anastomotic reconstruction with a “growth factor” (type 3 VR) or a “long” resection. In the latter setting (type 4 VR), if mesenteric root mobilization and lowering of the right liver are insufficient to compensate for the length of the VR (57), interposed graft reconstruction may be used, including an autologous venous or peritoneal (58), a cryopreserved homologous (59), a heterologous (60), or a prosthetic (61–65) graft. A recent study (66) reported the prognosis after reconstruction in 229 VRs (LE 4) and the median benefit to survival with segmental VR followed by a end-to-end anastomosis (usually planned). In this study, 129 patients underwent lateral VR followed by a direct suture (Group 1: 56%), 64 underwent a segmental VR followed by end-to-end anastomosis (Group 2: 28%) and 36 underwent VR followed by interposed graft reconstruction (Group 3: 16%). The surgical morbidity and mortality were comparable in all 3 groups. However, median survival was significantly different in the three groups: 27.6 months, 18.8 months and 13 months in groups 2, 1, and 3, respectively (66).
  If the venous splenoportal confluence is resected, the splenic vein territory is at risk of: (a) segmental portal hypertension (SPH) with gastric congestion; (b) varicose veins at the gastrojejunal anastomosis and pancreaticojejunal sites and esophageal varices with a late risk of upper GI bleeding; and (c) splenomegaly and thrombocytopenia, in case of prolonged survival (67, 68) (LE 4). In case of gastric congestion, reimplantation of the splenic vein is possible in the inferior mesenteric vein (IMV) (69) or the left renal vein (44, 54) (LE 4). However, reimplantation is not necessary if the resection has preserved the confluence between the splenic and left gastric veins and/or the IMV (70, 71) (LE 4).
5. Impact of the reconstruction technique on the long-term permeability of venous reconstructions.
  A recent meta-analysis has shown that reconstruction with interposition grafts (IG) influences the long-term permeability of venous reconstructions but not survival (65). This meta-analysis of 14 studies including 257 VRs with IG and 570 VRs without, showed that when venous reconstruction was performed with an IG, post-operative morbidity, mortality, and survival at 1, 3, and 5 years were comparable to those observed with other reconstruction techniques.
  However, the risk of venous axis thrombosis was significantly higher at 6 months (OR = 2.75; 95% CI = 1.32–5.73; *p* = 0.007) (65) (LE 3). This meta-analysis confirmed the study by Liao et al. (62) which showed no difference in survival in 65 VRs for PAC reconstructed with 29 prosthetic grafts vs. 36 direct end-to-end anastomoses. The median survival was 11 and 12 months, respectively, and 1 and 3-year survival rates were 36 and 4% vs. 36 and 9%, respectively (LE 4).
  In a study of 173 VRs, 3 factors, excluding local-regional recurrences, favored the development of secondary thrombosis: preoperative chemotherapy (53 vs. 9%; *p* < 0.0001), preoperative radiation therapy (35 vs. 2%; *p* < 0.0001), and surgical duration (618 ± 57 vs. 424 ± 20 min; *p* = 0.002) (72). In this study, patients with thrombosis were more likely to have received a prosthetic graft than patients with patent venous reconstruction (18 vs. 2.7%; *p* < 0.03; OR: 7.7). On multivariate analysis, operative time (OR: 1.01; 95% CI, 1.01–1.02) and prosthetic graft (OR: 8.12; 95% CI, 1.1–74) were independent predictors of thrombosis (LE4). In another study including 90 VR with different techniques of reconstruction, the rate of thrombosis was 18% (16/90) and varied according to the technique (73). All reconstructions with primary end-to-end anastomosis (*n* = 28) or transverse venorrhaphy (*n* = 9) remained patent while longitudinal venorrhaphy (LV: *n* = 17), patch venoplasty (pv: *n* = 17) and graft reconstructions (GR: *n* = 19) were all associated with significant rates of thrombosis (*p* = 0.001 vs. no thrombosis). The rates of thrombosis were 23% for LV (4/17), 29% for pv (5/17) and 37% for GR (7/19), respectively. In that study, neoadjuvant therapy did not influence the vascular permeability rate of venous reconstructions and long-term aspirin did not have a preventive effect (LE 4). In a North American series of 43 VRs (2007–2013), all patients received aspirin or low molecular weight heparin (LMWH) whatever the reconstruction technique (63) (Table 3). After a median follow-up of 13 months, the venous permeability rate was 91%. Four patients (9%), 2 on LMWH and 2 on aspirin, developed postoperative thrombosis detected after a median of 72 days (range, 16–238) (LE 3).
  There are no recommendations for the prevention of thrombosis by anticoagulation or longterm aspirin. A systematic review compared data from 8 studies using anticoagulation (AC+ group: aspirin, clopidogrel, heparin or warfarin; *n* = 266) and 5 studies without any “preventive” methods (AC– group: *n* = 95) (74). However, in the AC+ group, treatment compliance was only 50% and more grafts were interposed (30 vs. 2, Fisher's exact test: *p* < 0.001). The post-operative morbidity and mortality rates were comparable in both groups. The early mesenterico-portal thrombosis rates were not significantly different (AC+: 7%, vs. AC−: 3%, Fisher's exact test: *p* = 0.270) between the groups and this complication was associated with high mortality (8/20: 40%). Early mesenteric portal thrombosis rates were comparable after excluding interposed grafts (1 and 2%) (LE4).
  Finally, acute thrombosis is very rare in the immediate post-operative period. In a multicenter study of 406 VRs, only 7 patients developed acute thrombosis (1.7%) (38) (LE 4) and in a Japanese study of 197 VRs, only 3 patients developed acute thrombosis (1.5%) (75) (LE 4). Overall, the 1-year permeability rates ranged from 82 to 93% (61–63, 75) (Table 3) (LE 4). Conversely, late thrombosis is frequent, often associated with recurrence (75% after a median of 15 months), and accompanied by portal hypertension and ascites in 75% of cases (61, 72, 75, 76) (LE 4). Percutaneous insertion of a stent under fluoroscopic guidance can treat ascites and decrease the risk of death from recurrent bleeding (76) (LE 4).
6. Histological invasion of the resected vein (V+) is a marker of tumor aggressiveness (37, 77–83) (LE 4).
  6.1. The degree of the tumor/vein interface (TVI ≤ or > 180°) on high-quality CT is predictive of V+ and the grade of invasion (36, 78) (LE 4). In the study by Nakao et al. (36) (297 VR including 174 V+, 66%), the V+ rate was 51% (42/82) for unilateral venous contact (NAKAO PV-B), 74% (72/97) for bilateral venous contact (Nakao PV-C) and 93% (63/68) for complete stenosis (Nakao PV-D) (36) (LE 4). In another study by Tran Cao et al. (78) (98 VR), despite neoadjuvant therapy in ~80% of patients, the V+ rate was 69% (64/93 evaluated, including 42 media or intima invasions); the V+ rate was 29% in the absence of tumor/vein contact, 65% in the presence of a TVI ≤ 180°, 80% in case of TVI > 180° and 89% in case of venous occlusion (Area under the curve = 0.768) (LE 4).
  6.2. The prevalence of V+ is estimated in various ways, mainly because there is no standardized pathological protocol and due to “missing” results because the resected vein is not identified on the surgical specimen by the surgeon (especially in the case of lateral resection). V+ varies from <40% (81) to nearly 80% (82) or 100% (83) (LE 4). In a Japanese series of 160 VRs there were more V+ with distal pancreatectomy (29 V+/55 DP including 8 patients with celiac axis en bloc resection: 53%) than with PD (33 V+/105 PD: 31%) (*p* = 0.009) (37) (LE 4). In 2 recent meta-analyses on VR (79, 84), 39% (17–78% depending on the series included) (79) and 42% (84) of resected veins did not show any histological invasion (LE 3).
  6.3. The prognostic value of V+ “*per se*” is debated because the results differ among monocentric retrospective studies (85). In 2 retrospective monocentric studies involving more than 100 patients (229 and 136 VRs), V+ had no negative prognostic value “*per se*” (65, 86) (LE 4). However, in most observational studies (38, 50, 78, 82, 87–90) (LE 4) and in one meta-analysis (91) (LE 3), survival was reduced in patients with V+, and it was an independent negative predictive factor, including after neoadjuvant treatment (92) (LE 4). In a case-control study of VRs matched for venous invasion (81) (36 V+ patients vs. 66 V– patients), median overall survival (11.9 vs. 16.1 months; *p* = 0.01) and progression-free survival (7.4 vs. 10.9 months) were significantly reduced and there were more metastatic events (75 vs. 46%; *p* = 0.01) for V+ (LE 4). In a monocentric retrospective series of 90 segmental VRs (59% with neo-adjuvant treatment), V+ was observed in 58% (52/90 including 34/52 media or intima) (93); overall survival was reduced, although this was not statistically significant (14 vs. 21 months, *p* = 0.08), and recurrence-free survival was significantly altered, mainly due to locoregional recurrence (11.3 vs. 15.8 months, *p* = 0.03) (LE 4). In the meta-analysis published by Song et al. (79) (2000–2016−18 observational studies−5,242 pancreatectomies including 2,199 VRs (42%), and a V+ rate of 58% for 1,218/2,096 pathologic examinations of the venous wall) V+ had a significant independent negative impact on survival (HR = 1.88; 95% CI = 1.48–2.39; *p* < 0.001) (LE 3). In this meta-analysis, V+ was significantly associated with poorly differentiated tumors (*p* = 0.002), N+ (*p* < 0.001), perineural invasion (*p* < 0.001), R1 resection (*p* = 0.004), and recurrence (*p* < 0.001) (79). On the other hand, a recent study from the MD Anderson Cancer Center (94) in 127 patients including 114 (90%) who received neoadjuvant therapy, did not report any negative prognostic value for cancer cells at the vein edge, suggesting that transection of the SMV-PV through a macroscopically normal vein may be performed to minimize resected vein length with no negative effect on oncological outcomes. On the other hand, cancer invasion in the lumen was adversely associated with recurrence free and overall survival (*p* < 0.05) (94) (LE 4).
  6.4. The extent of tumor invasion (grades 1: adventitia, 2: media, and 3: intima) described by Nakao (18) is often poorly evaluated and the prognostic value of this feature is also debated (82, 87, 89, 90, 93), including after neoadjuvant treatment (92, 94) (LE 4). In the series by Roch et al. (93), the 3 grades of tumor invasion had no significant impact on overall or disease-free survival (14.4 vs. 15.5 vs. 7.4 months, *p* = 0.08 and 11.2 vs. 12.2 vs. 5 months, *p* = 0.59, respectively), although survival was very short in case of intra-luminal tumor invasion (however, the number of patients was low) (LE 4). Conversely, in the study by Addeo et al. (95), V+ was not associated with a significant reduction in overall median survival (20 vs. 27 months; *p* = 0.08) but invasion of the intima was found to be an independent predictor of poor survival (HR = 2.25; *p* = 0.0001) (LE 4).
    Finally, it is difficult to intraoperatively distinguish V+ from adventitious fibrotic adhesions secondary to peritumoural inflammation (96), particularly after neoadjuvant treatment. Although a desmoplastic reaction will result in negative pathologic examination of the resected vein, the benefit of a neoadjuvant strategy exceeds the risk of incomplete resection (15) (LE 2). In all studies, the survival of V– patients is comparable to that of patients with “standard” resection (97) (LE 4). However, a matched comparative study on small samples (98) (19 PD+VR with V-: 10 “upfront” VR/9 after chemoradiotherapy vs. 19 patients in the control group: 11 “upfront” VR/8 after chemoradiotherapy) reported that survival was better in patients who underwent VR and whose vein was V– than in patients who underwent standard PD, suggesting the benefit of systematic venous resection in the absence of any venous contact (LE 3).
7. Results of VR morbidity, mortality and survival.
  7.1. Many comparative monocentric studies have reported equivalent results for postoperative morbidity, mortality, and survival after PD with or without VR in the absence of neo-adjuvant treatment (i.e., upfront) (99, 100) (LE 4). Thus, in 2014, the International Study Group of Pancreatic Surgery (ISGPS) recommended “upfront” VR for borderline tumors (56) (LE 3), despite the potential benefit of multimodal treatment (28–31) (LE 2). Regarding morbidity, only one series of 127 VRs reported a high rate of postoperative bleeding with, surprisingly, one fourth of the patients requiring repeat laparotomy, usually for bleeding (101). However, the Comprehensive Complication Index (Supplemental Material 5) did not differ from that in 657 standard PDs (median score 8·7 vs. 8·7; *p* = 0·175) due to low 90-day mortality (3.1 vs. 3.3% (LE 4).
    Regarding survival rates, a Japanese monocentric comparative study including 375 patients, including 142 classified as “borderline resectable” who underwent upfront resection, showed that the rate of R0 resections was lower in case of VR (*n* = 91) (69 vs. 77%) including when preoperative CT scan demonstrated unilateral venous contact (Nakao Type B) (77). Moreover, the N+ rate was higher (80 vs. 65%) and the prognosis was poorer than that in patients with “clearly” resectable tumors (median **cancer-specific** survival: 14.4 vs. 24.4 months and median recurrence-free survival: 12 vs. 16.5 months; *p* = 0.0038). Survival was correlated with the severity of venous involvement observed on preoperative CT scan (Nakao Types B, C, or D: median specific survival 26, 12, and 16 months, respectively). Post-operative chemotherapy had a positive impact on cancer-**specific** survival regardless of the type of venous extension (Nakao Type B: 26 vs. 13 months; Type C: 27 vs. 8.6 months, *p* < 0.0001; Type D: 20 vs. 9.6 months, *p* < 0.0052), but compliance to treatment at 3 and 6 months was lower in case of venous involvement (57 and 45% vs. 73 and 55%, respectively) (77) (LE 4).
  7.2. Several comparative national surveys have reported conflicting results. They have mainly included VRs performed without neoadjuvant treatment.
    7.2.1. A survey performed in the United Kingdom (“UK Vascular Resection for Pancreatic Cancer Study Group”) was reported in 2 publications (102, 103) (LE 3). This survey included 1,070 patients, 840 who underwent standard PD and 230 with PD+VR. The rates of delayed gastric emptying (11 vs. 5%; *p* = 0.0007) and blood transfusion (32 vs. 22%; *p* = 0.002) were significantly higher in the PD + VR group, but hospital mortality and survival were comparable (18 months in both groups) despite a higher R1 resection rate in PD+VR (63 vs. 52%; *p* = 0.003; 71% for histologically positive veins).
    7.2.2. Three surveys conducted in North America (“American College of Surgeons—National Surgical Quality Improvement Program Database”) reported conflicting results on morbidity and mortality with VRs (104–106) (Table 4). The most recent study published in 2017 (106), was performed over a 14-month period in 43 institutions (Pancreatectomy Demonstration Project) and included 1,414 PDs: 1,162 standard (82%), 194 PD + VRs (14%), and 58 PDs with arterial resection (PD + ARs: 4%). Overall morbidity and surgical mortality were comparable in the 3 groups (standard PD: 44 and 1.5%; PD + VRs: 47 and 3.6%; PD + ARs: 51 and 3.6%, respectively; NS). However, venous resections were associated with a longer operating time, higher transfusion rates, more septic events, more deep venous thrombosis and a longer hospital stay (LE 4).
    7.2.3. An observational study performed from 2001 to 2012 in Japan included 937 PDs and compared the results of 435 CPD+VRs (46%) to 502 standard PDs (54%) (107). The mortality and morbidity rates of PD+VR were comparable to those observed after standard PD (respectively: 2% at 90 days in both groups and 21 vs. 19.5%). Overall survival was comparable (HR = 1.16; *p* = 0.20) but median survival was significantly different (PD+VR: 18.5 vs. standard PD: 25.8 months; *p* < 0.001). This study suggests that venous resection should be limited to patients with no arterial contact on preoperative imaging (median survival: 30 vs. 18.6 months). Adjuvant chemotherapy was found to be an independent predictor of survival (patients with borderline resectable tumors with PV/SMV involvement had a median survival of 29.7 months; HR = 3) (LE3).
    7.2.4. A study performed in France by the French Association of Surgery included 1,399 resections (1,325 PDs and 74 TPs) performed from 2004 to 2009, including 997 standard resections and 402 VRs (29%) (41). Post-operative morbidity and mortality rates were comparable, but survival was significantly reduced in the case of venous resection, including in the subset of the R0N0 patients. VRs were associated with larger (*p* < 0.001) and more often undifferentiated (*p* = 0.004) tumors. Lymph node invasion (*p* = 0.042) and R1 resections were also more frequent (*p* < 0.001). Overall morbidity and post-operative mortality (PD+VR: 5 vs. 3%; *p* = 0.16) were comparable. The median and 3-year survival rates of PD+VR were significantly reduced [21 months and 31% vs. 29 months and 44%, respectively (*p* = 0.0002)]. Multivariate analysis showed that VR was a negative prognostic factor (HR = 1.75; 95% CI = 1.28–2.40; *p* = 0.0005). However, VRs after neoadjuvant treatment were associated with a better prognosis (HR = 0.52; 95% CI = 0.29–0.94; *p* = 0.031). In the PD+VR group, three factors were found to have independent negative prognostic value in multivariate analysis: the N+/N ratio, regardless of the cut-off (0.1 and 0.2: *p* = 0.093; ≥ 0.3: *p* = 0.0098), R1 resection (*p* = 0.010) and segmental VR (*p* = 0.016). Finally, adjuvant chemotherapy was found to be an independent predictive factor of a good prognosis (HR = 0.55; 95% CI = 0.35–0.85; *p* = 0.006) (LE 3), as previously reported by Yamada et al. (77) (LE 4).
  7.3. Four meta-analyses were selected for a comparison of VR results with those observed after “standard” resection (84, 91, 108, 109) (Table 5).
    7.3.1. The meta-analysis including the largest number of patients, and published in 2016 (84) (27 studies−9,005 patients including 1,587 PD+VR) reported an increased risk of postoperative mortality (“risk difference” (RD) = 0.01; 95% CI = 0.00–0.03; *p* = 0·02) and resection R1/R2 vs. R0 (RD = 0.09; 95% CI = 0.06–0.13; *p* < 0.001) in case of PD+VR. In addition, survival at 1, 3, and 5 years was significantly reduced (respectively: HR = 1.23; 95% CI = 1.07–1.43; *p* = 0.005; HR = 1.48; 95% CI = 1.14–1.91; *p* = 0.004 and HR = 3.18; 95% CI = 1.95–5.19; *p* < 0.001). Median overall survival was 14.3 months in the PD+VR group vs. 19.5 months in the standard PD group (*p* = 0.063). This meta-analysis concluded that neo-adjuvant treatment was recommended in the setting of planned VR (LE 2).
    7.3.2. The most recent meta-analysis, published in 2017 (108) (16 studies−4,145 patients including 1,207 PD+VR) confirmed the results of the previous study on the increased risk of post-operative mortality (OR = 1.72(1.02–2.92); *p* = 0.04) and R1 resection (OR = 1.59(1.35–1.86) *p* < 0.0001) as well as the significant reduction in 5-year survival (HR = 0.20(0.070.55); *p* = 0.020) in the VR group. Patients had larger tumors (*p* = 0.030) and a higher perineural invasion rate (*p* = 0.009). This meta-analysis concluded that “upfront” venous resection was not cost-effective and that indications for surgery were needed (LE 2).

**Table 2:** Frequency of venous resections associated with distal pancreatectomies in the literature.

**References**	n VR all PR*	**VR + DP**	**% VR + DP**
Nakao et al. (36)	297	15	5%
Okabayashi et al. (37)	160	55	34%
Ramacciato et al. (38)	406	87	21%
Rosso et al.^¥^ (39)	–	18	32%
Paye et al.^£^ (40)	402	33	7.5%

PR*: pancreatic resections (PD, DP, TP).

£ *1,399 patients included in a French multicentre survey (41); 402 patients with VR; 271 distal pancreatic resections: VR: 33/271: 12%*.

¥ *Series of distal pancreatectomies using the RAMPS technique*.

**Table 3:** Permeability of venous reconstructions after resection for cancer; results of literature (EEA, end to end anastomosis; PTFE, poly tetra fluoro ethylene graft).

**References**	**VR**	**Reconstruction**	**Follow-up average (months)**	**Permeability (% and median duration)**	**Thrombosis during hospitalization**	**Thrombosis**
Chu et al. 2010 (61)^*^	33	PTFE	14	76% 21 months	3	5
Krepline et al. (63)^**^	43	all types	13	91%	–	4
Liao et al. (62)^£^	36	EEA	–	6/12 months 94–86%	–	5
	29	PTFE	–	6/12 months 88–83%	1	5
Fuji et al. (75)^$^	197	EEA	–	–	3	18

* *US review (1994–2009); (PTFE—median diameter: 12 mm (8-20); “ringed” in 73% of cases). No graft infection. Mortality: 2 patients (6%) including 1 of 3 patients with early thrombosis*.

** *Suture (7, 16%), venous saphenous “patch” (9, 21%), terminal anastomosis (13, 30%), jugular graft (14, 33%); all patients received aspirin or low molecular weight heparin (LMWH); 4 thromboses: 2 on LMWH and 2 on aspirin within a median of 72 days (16-238)*.

£ *3-center study in China (2007–2012); 76 RV (65 for PDAC). Thrombosis after PTFE on day 4 was treated with thrombolysis (heparin + urokinase). The delay for late thrombosis was 3, 3, 5, 5, 11, and 17 months in the PTFE group and 4, 5, 8, 12, and 22 months in the TA group. Morbidity: 29% PTFE vs. 33% TA; mortality: 3% PTFE vs. 7% TA (NS despite PTFE for larger tumors (p = 0.016), longer operating time (p < 0.001) and greater bleeding (p = 0.04). There was no graft infection. There was no difference in survival for the 65 PDACs (29/65 PTFE and 36/65 TA; median 11 vs. 12 months; survival at 1 and 3 years: 36 and 4 vs. 36 and 9%, respectively)*.

$ *Series conducted in Japan: 197 VRs (197/810 pancreatectomies; 2000–2014); controlled permeability every 4–6 months to assess the rate of severe secondary anastomotic stenosis (≥70% of the caliber; AUC = 0.83); 3 acute thromboses after immediate surgery: 2 reoperations (1 reattempt; 1 venous graft); 1 conservatively treated). Excluding the 21 stenoses related to early neoplastic recurrence, 18 patients had severe, symptomatic secondary stenosis in 16 cases (refractory ascites: 9, encephalopathies: 4, and gastrointestinal hemorrhages: 7, including 2 treated with a stent and 1 by mesocaval shunt). The multivariate analysis showed independent factors for the occurrence of severe stenosis: the surgical duration (≥520 min; HR = 15.24; 95% CI: 3.75–104.4; p < 0.001) and the resected vein length >3 cm (HR = 5.96; 95% CI: 1.8–22.7; p = 0.003). This study suggested that an autologous graft could reduce this rate*.

**Table 4:** Morbi-mortality of venous resections (VR) during pancreatico-duodenectomy (PD): North American surveys (“American College of Surgeons—National Surgical Quality Improvement Program Database”).

**References**	**Period**	***n***	**VR**	**Morbidity**	**Mortality**
Castleberry et al. (104)^*^	2005– 2009	3,582	281 (8%)	40 vs. 33%, OR = 1.36; *p* = 0.02	5.7 vs. 2.9%, OR = 2.1; *p* = 0.008
Worni et al. (105)^¥^	2000– 2009	10,206	412 (4%)	OR = 1.36, *p* = 0.008	6 vs. 2%^¥^, OR = 4.32; *p* < 0.001
Beane et al. (106)	14 months	1,414	194 (14%)	47 vs. 44%, NS	3.6 vs. 1.5%, NS

* *Adjusted post-operative mortality risk*.

¥ *Adjusted propensity scores; higher risk of intraoperative complications: OR = 1.94, p = 0.001; comparable mortality and hospitalization times for all data sets but the mortality shown in the table is observed (paradoxically) in high volume hospitals*.

*All 3 surveys reported longer operating times and higher transfusion quantities*.

**Table 5:** Meta-analyses of venous resections (VR) during pancreaticoduodenectomies for cancer.

**References**	**Studies**	**Patients**	**VR**	**Morbidity**	**Mortality**	**3-years survival**	**5-years survival**
Zhou et al. (109)^*^	19	2,247	661	OR = 0.95 *p* = 0.67	OR = 1.19 *p* = 0.48	–	OR = 0.57 *p* = 0.06
Yu et al. (91)^¥^	22	2,890	794	NS	NS	NS	OR = 0.69 *p* = 0.03
Giovinazzo et al. (84)^£^	27	9,005	1,587		RD = 0.01 ^£^ *p* = 0.02	HR = 1.48 *p* = 0.004	HR = 3.18 *p* < 0.001
Bell et al. (108)^$^	16	4,145	1,207		OR = 1.72 *p* = 0.04	–	HR = 0.20 *p* = 0.020

* *Less pancreatic fistulas: OR = 0.53 (IC 95%: (95% CI: 0.35–0.79; p = 0.002)*.

¥ *Less pancreatic fistula; FP: p = 0.01; VR group: larger tumors (p < 0.001), N+ (p = 0.03), R1 (p < 0.001); R1 independent negative factor for survival at 2 years (OR = 2.93, p < 0.001) and 5 years (OR = 4.25; p < 0.00002). Histological invasion of the vein: independent factor of poor prognosis (OR = 0.29; p = 0.004)*.

£ *RD, risk difference; VR group: resection R1/R2: RD = 0.09 (95% CI: 0.06–0.13; p = 0.001) Median overall survival of the VR group: 14.3% vs. 19.5 months; p < 0.063*.

$ *VR Group: resections R1: OR = 1.59 (IC 95%: 1.35–1.86); p < 0.0001): larger tumors (p = 0.030); higher perineural invasions rate (p = 0.009)*.

### Arterial Resections (AR)

#### “What Are the Indications of Arterial Resection?”

##### Recommendations

Due to their complexity and specificities, planned PDs with arterial resection (excluding SMA) must be discussed in multidisciplinary team (MDT) meetings in tertiary referral centers (expert opinion).

1. A PD with planned arterial resection (except for SMA) may be proposed in selected patients with stable tumors or after tumor response to neoadjuvant therapy. This must be evaluated according to the location of the tumor and the type of arterial extension (Grade B):
  - in case of accessory right HA in the vicinity of the tumor, preoperative embolization followed by “en bloc” resection is recommended (expert opinion),
  - in case of right HA—total liver: resection after neoadjuvant therapy, including arterial reconstruction (using graft interposition if needed) may be proposed (expert opinion),
  - in case of invasion of a short segment of the common HA (invasion of the origin of the GDA): resection after neoadjuvant therapy with arterial reconstruction, may be proposed (expert opinion).
2. In case of SMA invasion, neoadjuvant therapy is recommended, followed by laparotomy with dissection and biopsy of peri-arterial tissues in case of tumor stability or tumor response. If the frozen section examination is positive, a PD with arterial resection is not recommended (Grade C).
3. In case of distal PDAC with invasion of the celiac axis, neoadjuvant therapy is recommended. In case of stabilization or tumor response, a distal pancreatectomy with celiac axis resection without arterial reconstruction may be proposed after radiologic embolization of the CA branches (expert opinion).

##### Comments

1. The invasion of the common hepatic artery (CHA) or the gastroduodenal artery (GDA) at its origin, of the superior mesenteric artery (SMA), or the celiac axis (CA) is usually considered as a contraindication for resection due to the risks of both morbidity and mortality, and poor oncological results (56, 110–113) (LE 3). A study in Japan (77) including 137 resectable tumors and 142 tumors classified as “borderline” on imaging (91 PV+, 21 CHA+, and 30 SMA+) showed that in the absence of neaoadjuvant therapy patients with histological invasion of CHA (*n* = 21) or SMA (*n* = 30) were more often N+ (resectable: 65%; VP+: 80%; CHA +: 86%; SMA +: 93%; *p* < 0.001) and had fewer R0 resections (resectable: 105 R0/32 R1 (77%); PV+: 64 R0/27 R1 (69%); CHA +: 10 R0/11 R1 (48%); SMA +: 11 R0/19 R1 (37%); *p* < 0.0001) (LE 3).
  The report from the French Association of Surgery (41) (2004–2009) showed that in France AR was performed during pancreatectomy for PDAC in 2% of cases (37/1670, 27 + VR) with a morbidity rate of 54%, a 30-day mortality rate of 8% and a 3-year survival rate of 8% (median: 12.7 months, median survival rate without recurrence, 7 months). In a VR study in this series, AR significantly increased mortality (RR = 2.09; 95% CI = 0.99–4.38; *p* = 0.05) (LE 4).
  A meta-analysis published in 2011 selected 26 studies (adding up to 366 ARs vs. 2,243 non-AR pancreatectomies) including only 5 studies with SMA resection all involving fewer than 30 patients (114). This meta-analysis reported: (a) a significantly increased risk of morbidity (median: 54%) and surgical mortality (median: 12%) (OR = 5; 95% CI = 2.69–9.45; *p* < 0.0001; I^2^ = 24%); (b) a significant reduction in survival at 1 year, including after exclusion of post-operative mortality (49%; OR = 0.49; 95% CI = 0.31–0.78; *p* = 0.002; I^2^ = 35%), at 3 years (8%; OR = 0.39; 95% CI = 0.17–0.86; *p* = 0.02; I^2^ = 49%), with no survivors at 5 years; and (c) a significantly higher operative mortality (with heterogeneous delays: in-hospital, 30 to 90 days) than pancreatectomy with VR (OR = 8.87; 95% CI = 3.40–23.13; *p* < 0.0001; I^2^ = 5%) which was associated with a significantly higher 1-year survival (OR = 0.50; 95% CI = 0.31–0.82; *p* = 0.006; I^2^ = 40%). AR still had a negative prognostic value when mortality was adjusted for tumor size, R1 resection and synchronous VR. However, in this meta-analysis, most of the included studies reported patients receiving upfront resections (LE 2).
  Since this meta-analysis, several retrospective mono-centric studies including small numbers of patients have reported AR results (mainly common HA or celiac axis, more rarely SMA) (44, 117–119) (LE 4). A recent systematic review (115) (2000–2016) of 13 studies including 70 patients undergoing pancreatectomy with SMA resection, which is rarely performed (out of 10,726 undergoing pancreatectomy) concluded that there was no evidence to support SMA resection. Indeed, in the 25 patients with available individual patient-level outcome data, perioperative morbidity ranged from 39 to 91%, the mortality rate was 25% and median survival was only 11 months (LE 4).
  However, the increasing use of neoadjuvant therapy protocols has increased the pool of selected patients who are candidates for “secondary” resection despite an initial suspected arterial invasion (116).
  Both the Mayo Clinic group (24, 120) and Bachellier et al. (121) reported more than 100 ARs (LE 4). In 2018, the Mayo Clinic group reported results in 111 patients who underwent pancreatectomy with AR [HA (*n* = 60), celiac (*n* = 49), SMA (*n* = 15), multiple ARs (*n* = 15)] including 55% with reconstruction and 51% with simultaneous VRs (120). Most cases were planned (77%) and were performed after 2010 (78%). Overall 90-day major morbidity (≥ grade III) and mortality were 54 and 13%, respectively. Post-pancreatectomy hemorrhage (mainly related to POPF) was associated with major morbidity (OR 5.1, *p* = 0.005), reoperation (OR = 23.0, *p* = 0.004), ICU (OR 5.5, *p* < 0.001), readmission (OR 2.6, *p* = 0.004) and increased mortality (OR 6.1, *p* < 0.001). Median survival was 28.5 months. A significant decrease in mortality was observed after 2010 (9 vs. 29%, *p* = 0.02) (120). In 2019, the same group published the results of “total neoadjuvant therapy” (i.e., systemic chemotherapy followed by chemoradiation) in 123 (63%) BR and 71 (37%) LA PDAC resected between 2010 and 2017 (LE 4) (24). Sixty-four (33%) patients did not undergo AR and 50 patients underwent simultaneous VR and AR (26%). Overall, 69 patients (36%) had major complications (including 34/64 ARs; 53%). Overall 90-day mortality was 6.7% with 13 deaths including 8 patients (62%) who underwent combined VR/AR resection. Only 62 (32%) patients received adjuvant chemotherapy, as also reported by Loveday et al. (39%) (119) (LE 4). Median and 3-year recurrence free survival and overall survival rates were 23.5 and 58.8 months, and 32 and 62%, respectively. Multivariate predictors of RFS and OS were: ≥ 6 chemotherapy cycles (HR = 0.45; *p* < 0.001), optimal CA19–9 response (HR = 0.49; *p* = 0.01), and major pathological response (HR = 0.16; *p* < 0.001).
  Bachellier et al. (121) reported 118 pancreatectomies (51 PD, 18 TP, and 49 DP) with ARs [CA (50), HA (29), SMA (35), and other segments (4)] including 85% with reconstruction and 89% with simultaneous VR (LE 4). Overall mortality and morbidity were 5.1 and 41.5%, respectively. The rates of R0 resection and pathological invasion of venous and arterial walls were 52, 74, and 58%, respectively. Median overall survival after resection was 13.7 months. In multivariate analysis, R0 resection (HR: 0.60; *P* = 0.01) and venous invasion (HR: 1.67; *P* = 0.04) were independent prognostic factors.
  On the other hand, the Heidelberg group reported that they described as a more conservative approach to the major arterial axes, in particular the SMA:
  - a first study published in 2016 (122) reported that, out of 65 ARs performed in 1,828 patients, only 18% received neoadjuvant therapy (65/1828: 3.5%; 12 PDs, 8 distal pancreatectomies and 45 TPs; *p* < 0.001). In that study, ASA grade III-IV vs. grade I-II scores were predictive of hospital mortality (OR = 2.65; 95% CI = 1.34–5.52; *p* = 0.007). AR was not found to be an independent factor of mortality “*per se*,” but multivariate analysis of hospital mortality factors identified TPs, with a 90-day mortality rate of 16%, and operating times as confounding factors with a high relative risk of death (TP: OR = 2.37,; 95% CI = 1.22–4.7; *p* = 0.012; operating times 300 min. −419 vs. < 300 min.: OR = 4.99; 95% CI = 1.33–32.45; *p* = 0.038; operating times ≥ 420 vs. < 300 min.: OR = 11; 95% CI = 3.2–70.4; *p* = 0.001) (LE 4).
  - a study published in 2017 by the same group (123) described radical tumor removal by sharp dissection along the CA and the SMA with complete dissection of all soft tissue between both arteries and superior mesenteric/portal vein (“TRIANGLE operation”). In case of positive frozen section(s) of the arterial sheaths, “non-resection” and palliative treatment were indicated. This study included a consecutive series of 15 patients. The R0 resection rate (1 mm) was 40% (6/15) in patients who had pancreatectomy with “arterial sparing” resection (LE 4).
2. Three additional situations can be distinguished and in each of these 3, AR must be planned:
  2.1 First, anatomical variants of HA: “Right” HA arising from the SMA during a planned PD for a “clearly” resectable tumor:
    - The HA may be “accessory”: recent data suggest that preoperative embolization by interventional radiology followed several days later by “en bloc” resection may be performed with no significant risks of liver/biliary ischemia due to development of intrahepatic arterial shunts (124) (LE 4). A systematic review has shown the feasibility and lack of morbidity and mortality of this strategy (125) (LE 4). This strategy avoids opening the accessory tumor/HA interface, which can be exposed with the risk of tumor spillage in case of RHA preservation (126) (LE 4).
    - The HA can perfuse the “total liver” (Michell type 9: 1–5%): in this rare setting, it requires reconstruction of any type [direct anastomosis, by an interposed “reversed” saphenous vein graft (44, 53, 117) (LE 4) or reversal of the splenic artery (127) (LE 4)] to ensure vascularization of the biliary tree and the hepatico-jejunostomy following PD. HA should be reconstructed before continuing pancreatic resection to avoid any liver ischemia, particularly when an associated venous resection is needed.
  2.2 Second, PD and resection of modal HA:
    Most PDs with AR reported in the literature included resection of a short segment of the common HA, usually with reconstruction. Only one Japanese study by Miyazaki et al. (128) (LE 4) reported 20/21 patients who underwent HA resection without reconstruction. Twelve of these patients had received preoperative embolization of the common HA (CHA) for collateral vessel formation. In this short series there was no relevant specific morbidity, except for a temporary postoperative increase of liver enzymes.
  2.3 Third, distal pancreatectomy (DP) with celiac axis resection (DP-CAR), for PDAC of the body and tail with invasion of the CA or the origin of the CHA:
    The “Appleby” procedure was initially described for the resection of gastric cancers invading the celiac area. In the 1970's, Nimura et al. (129) described this technique for body and tail PDACs and showed improved survival compared to standard DP (129, 130). In the 1990's, Hishinuma et al. modified the technique by preserving the stomach (“modified Appleby”) (131) (LE 4).
    The principles of the intervention are to: (a) increase the rate of R0 resectability; (b) ensure lymphatic clearance around the CA and its branches; (c) preserve the collateral circulation from the SMA and pancreaticoduodenal arcades (PDA) to the liver, the biliary tract and the stomach, and (d) avoid any arterial reconstruction with subsequent anastomotic complications.
    This procedure is contraindicated when the CA is invaded at its origin on the aorta or if the GDA is invaded (132–134) (LE 4). Indeed, DP-CAR requires a tumor-free and patent GDA to ensure “reverse flow” vascularization of the liver and bile ducts from the SMA through the PDA and GDA (12) (LE 2) and, for many authors, the use of preoperative occlusion of CHA or, at the best, the 3 branches of the CA to favor development of arterial collaterals thus reducing the risk of bile ducts and gastric ischemia (135, 136) (LE 4). Embolization should be performed 1–2 weeks before resection (137, 138) (LE 4). This procedure, which avoids any arterial reconstruction, remains controversial. Some authors prefer reconstruction in case of insufficient flow during an intraoperative “Doppler” control (139, 140) (LE 4). Embolization is not effective in the case of “total liver” HA arising from the SMA, which requires reconstruction (137, 138) (LE 4).
    2.3.1 Monocentric studies:
      Several monocentric, mainly Japanese, studies with small groups of patients, have been published in the past 10 years and were included in two recent systematic reviews, with reported median survivals ranging from 10 to 26 months and 5-year survival rates of 20% (97) (LE 4).
      - A single-center study conducted in North America (139) included 11 patients who underwent preoperative chemoradiotherapy and showed that median recurrence-free survival was only 21 weeks despite a 90% R0 resection rate and an overall median survival rate of 26 months (LE 4).
      - A single-center series performed in Japan (135) included 80 consecutive DP-CARs (19982015), and reported rates of Clavien-Dindo ≥ III morbidity, pancreatic fistula, and ischemic gastropathy of 41% (*n* = 33), 58% (*n* = 47), and 25% (*n* = 20), respectively. Post-operative mortality was 5% (*n* = 4). The survival rate at 1, 2 and 5 years was 81, 57, and 33%, respectively, and median survival was 30.9 months. The survival rate was significantly higher in 12 patients who received neoadjuvant treatment (100, 90, and 79% vs. 78, 51.5, and 27%, respectively, in the 68 patients who received up front surgery; *p* < 0.0001) (135) (LE 4). Preoperative CHA embolization was routinely performed (median: 7 days before surgery: 1–16), and since 2007 embolization of the left gastric artery has been added to limit the risk of ischemic gastropathy (unchanged rate) (LE 4).
      - The rate of ischemic gastropathy was 10% in a Japanese series including 50 patients (11 with synchronous VR) (136) (LE 4). Twenty-six of the 50 patients received preoperative therapy (14 with chemotherapy, 11 with chemoradiotherapy) and the remaining 24 underwent upfront surgery. Twenty-two patients who had early division of the celiac axis with emergence of the left gastric artery (LGA) before the trunk of the hepatic and splenic arteries and had a distance between the LGA emergence and carcinoma >10 mm underwent a “modified DPCAR.” With this technique the CA is divided just below the LGA emergence. Left gastric artery resection (and a combination of left inferior phrenic artery resection) was a significant risk factor for ischemic gastropathy. Twenty-eight patients (56%) completed the planned adjuvant chemotherapy. The postoperative comparison at 2 and 3 months demonstrated higher nutritional values in patients who underwent LGA-preserving DP-CAR than those with LGAresecting DP-CAR. In this study, the R1-resection rate declined from 58% during the upfront strategy period to 19% during the neoadjuvant therapy strategy period (*P* = 0.005).
      Another recent retrospective study (141) compared the outcomes of patients receiving (*n* = 11) or not (*n* = 9) various regimens of neoadjuvant chemotherapy (mainly GEM-nab-PTX). Despite the small number of patients, those who received neoadjuvant chemotherapy had significantly less arterial invasion (*p* = 0.025), lymphatic invasion (*p* < 0.0001), and vascular invasion (*p* = 0.035) with significantly higher recurrence-free and overall survival rates.
      - A retrospective study in Japan (142) including 50 patients defined a prognostic score based on the following independent negative prognostic factors: intraoperative blood loss (≥940 mL, HR = 25; *p* = 0.0003) and 3 biological factors including preoperative thrombocytopenia (<150 x 109/L; HR = 7.4; *p* = 0.0043), CRP rate (≥0.4 mg/dL; HR = 7; *p* = 0.0018), and CA19-9 rate (≥300 U/mL; HR = 8; *p* = 0.0053). These 3 preoperative biological factors were assigned 1 point each. The total score was predictive of survival: with a score of 0 (26 patients) “disease-specific” 1- and 5-year survival was 96 and 49%, respectively, and median survival was 50.6 months. With a score of 1 (15 patients) 1-year survival was 87% (5 years: NA) and median survival was 22.3 months. Patients with a score of 2 or 3 had a 1- and 5- year survival of 33 and 0%, respectively, and a median survival of 7.7 months (LE 4).
      - A single-center, case-controlled study in North America (Johns Hopkins Hospital−2004–2016) (143) compared data from 17 patients (including 11 operated on in 2014 and 2015, 9/11 after neoadjuvant therapy) with data from 51 DP (1:3). The most common neoadjuvant therapy was Folfirinox (80%). Although the procedure was longer than DP without CA resection (404 vs. 309 min; *p* = 0.003), there was no significant difference in blood loss, overall morbidity, pancreatic fistula rate, length of hospital stay, surgical mortality, or readmission rates. The R0 resection rate was 82% in the DP-CAR group vs. 92% in the DP group (*p* = 0.35). Median overall survival was 20 months in the DP-CAR group vs. 19 months in the DP group (*p* = 0.76) (LE4).
    2.3.2 Multicentre studies:
      - A 14-month multicentre North American College of Surgeons-National Surgical Quality Improvement Program (ACS-NSQIP) Pancreatectomy Demonstration Project included 822 DPs from 43 hospitals (144). Twenty patients who underwent DP-CAR (“modified” Appleby; 2.4%) recruited in 16 centers (obviously a limitation for this study) were compared to 172 patients who underwent DP who were matched for age, sex, BMI, albumin blood level, ASA score, pancreatic consistency, main pancreatic duct diameter, and pathology (60% PDAC).
        The procedure was longer for DP-CAR (median 276 vs. 207 min; *p* < 0.01) and the rates of postoperative acute renal failure (10 vs. 1%; *p* < 0.03) and 30-day mortality (10 vs. 1%; *p* < 0.03) were also significantly higher (LE 3).
      - A multicentre comparative study in Japan (7 centers; 2001–2012) (145) included 395 patients: Group 1 (323 DP with splenectomy) and Group 2 (72 DP-CAR). Ninety-three percent of Group 1 patients had a “resectable” tumor while group 2 patients had borderline or locally advanced tumors at presentation. Post-operative morbidity was significantly higher in Group 2 (63 vs. 47%; *p* = 0.017) and the overall median survival was shorter (17.5 vs. 28.6 months; *p* = 0.004). In Group 2, 61/72 patients received adjuvant chemotherapy (85 vs. 20% in group 1). Overall median survival was longer in these patients than that in the 65 patients in Group 1 (65/323 = 20%) who underwent R1 resection (21.9 vs. 16.7 months; *p* = 0.024). This result suggests that: (a) DP-CAR is indicated in case of a high probability of R1 resection with standard PD; and (b) adjuvant CT is beneficial after DP-CAR (LE 3).
      - A multicentre retrospective European study was published in 2018 (146) (LE 3). This study included 68 patients who underwent surgery in 20 institutions from 2000 to 2016, and reported 53% R0 resection, 25% major morbidity, 21% grade B/C pancreatic fistula, and a 16% mortality rate. Overall, 82% of the patients received neo-adjuvant or adjuvant chemotherapy. Median survival in patients with PDAC was 18 months (95% CI = 10–37). Preoperative CA embolization was not associated with a lower risk of ischemic complications.
    2.3.3 Two systematic reviews were published in 2016:
      - The first included 19 studies (1975–2014) and 240 patients (147) (LE 3). Only 50% (0–100%) of the patients received neoadjuvant treatment depending on the study period and none survived after 5 years in the absence of neoadjuvant therapy. The rate of preoperative HA embolization ranged from 0 to 50%. CA resection was associated with VR in 38% of the cases. Clavien-Dindo III-IV morbidity was 27%, “ischemic” morbidity (cholecystitis, gastric perforation) was 10%, and 90-day mortality ranged from 0 to 18%. The R0 resection rate was 74%. Half of the patients received adjuvant therapy (range: 30–86%). Median survival was 14 months (9–25 months) and 18 months, respectively, for pre- and post-operative treatment.
      - The second study included 18 studies performed up to 2014 (148) (LE 3) and provided the following additional data: (a) a 11.5% vascular reconstruction rate; (b) a significantly higher frequency of delayed gastric emptying (HR = 5.67); (c) a comparable pancreatic fistula rate; (d) pain relief in 89% of patients; and (e) a 37% rate of post-operative diarrhea due to transit acceleration. This review concluded that despite longer surgery, a higher risk of transfusions, a 10% re-intervention rate, higher morbidity (HR = 2.1), and higher but not significant surgical mortality (HR = 1.8), survival with DP-CAR at 1, 2, and 3 years (65, 30, and 19%, respectively) was comparable to DP without CA resection [HR = 1.36; (95% CI = 0.997–1.850). Median and mean survival rates were 24 months (95% CI = 18.26–29.98) and 17 months (95% CI = 13.52–20.48), respectively.

ARs are therefore very rarely indicated, often associated with venous resection (24, 97, 120, 121) (LE 3), and must be “planned” since they require routine neoadjuvant treatment and frequent preoperative arterial embolization. Surgery should always begin with an “artery first” approach (34, 35) to accurately evaluate any persistent arterial involvement confirmed by frozen section examination (34, 35, 116). Forty (121) to 70% (24, 119) of patients do not receive postoperative chemotherapy (primarily single agent gemcitabine) due to postoperative morbidity and prolonged recovery time and there are few data on patient quality of life. Interestingly, a recent study from the MD Anderson Cancer Center (149) including 127 patients who received neoadjuvant treatment before PD (including vascular resection in 58 (46%); VR = 44, AR = 3, both = 11) reported that: (a) all patients experienced at least a transient skeletal muscle, visceral fat and subcutaneous fat loss; but (b) a relative increase in skeletal muscle (HR = 0.50) and albumin (HR = 0.57) during the first postoperative 12-months were associated with improved overall survival. This suggests that persistent postoperative skeletal muscle loss may represent an early marker of poorer outcomes.

## Conclusions

PD with venous resection improves survival compared to no resection, especially with R0 resection. Mortality and morbidity are higher in PD with venous resection than in PD without vascular resection. PD with upfront venous resection has a poorer oncological results (increased risk of R1 resection, poorer survival) than PD with venous resection after neoadjuvant treatment. PD with arterial resection is associated with increased morbidity and mortality (compared to PD with venous resection) and has not been shown to be beneficial. A distal splenopancreatectomy with celiac axis resection is associated with increased morbidity and mortality and the oncological benefit of this approach has not been clearly demonstrated.

Today, literature provides more support for neoadjuvant therapy in the management of pancreatic cancer. Waiting for RCTs results including clearly resectable tumors, neoadjuvant therapy and a complete R0 resection in all patients who require planned vascular resection with (or without) reconstruction should be the goal. Such patients should be treated by an experienced team in both preoperative/neoadjuvant therapy and vascular resection at the time of pancreatic resection. Such expertise is not available at every centers, which makes another strong case for the regionalization of complex cancer care that involves multiple treatments.

## Author Contributions

JD and AS contributed equally to the design and implementation of the research, to the analysis of the results, and to the writing of the manuscript.

### Conflict of Interest

The authors declare that the research was conducted in the absence of any commercial or financial relationships that could be construed as a potential conflict of interest.
