# Supplementary material for: Vascular Resection for Pancreatic Cancer: 2019 French Recommendations Based on a Literature Review From 2008 to 6-2019
Source: Front Oncol. 2020 Feb 4;10:40. doi: 10.3389/fonc.2020.00040 (PMC7010716; doi:10.3389/fonc.2020.00040)
Supplement: Supplementary file 1 [file Data_Sheet_1.docx]

**Supplemental material - Methodology.**

**a) Key-words used for literature search**

Pancreatic ductal adenocarcinoma (PDAC), pancreatic adenocarcinoma and vascular resection, and venous resection, and arterial resection, pancreaticoduodenectomy (PD), distal pancreatectomy (DP), total pancreatectomy (TP), and vascular resection ; Appleby procedure ; Vascular resection and neoadjuvant treatment.

b) **Level of evidence and GRADE** **of recommendations** (Grading of Recommendations Assessment, Development, and Evaluation system)

| Level of evidence |  | GRADE |
| --- | --- | --- |
| Level 1 | • Large RCT (with low potential for bias)  • meta-analyses of well-conducted RCT without heterogeneity | A : Strong scientific evidence (efficacy with a substantial clinical benefit) ; strongly recommended |
| Level 2 | • Small RCT (or large RCT with a suspicion of bias *ie* low methodological quality)  • meta-analyses including such trials  • well conducted non randomized comparative studies | B : Strong or moderate evidence for efficacy but with a limited clinical benefit, generally recommended |
| Level 3 | • prospective cohort studies  • case–control studies | C : low level of evidence |
| Level 4 | • comparative studies with bias  • retrospective studies  • case series |  |

• experts opinions should be considered as level 5

References :

• Atkins D, Briss PA, Eccles M, Flottorp S, Guyatt GH, Harbour RT*, et al.* Systems for grading the quality of evidence and the strength of recommendations II: pilot study of a new system. BMC Health Serv Res 2005; 5(1) : 25.

• OCEBM Levels of Evidence Working Group*. *The Oxford 2011 Levels of Evidence*. Trans Durieux N, Pasleau F, Howick J. Oxford Centre for Evidence Based Medicine.

hbp://www.cebm.net/ index.aspx?o=5653

• http://www.has- sante.fr/portail/upload/docs/application/pdf/analiter at.pdf

•[https://www.has-sante.fr/upload/docs/application/pdf/2013 06/etat_des_lieux_niveau_preuve_gradation.pdf](https://www.has-sante.fr/upload/docs/application/pdf/2013-06/etat_des_lieux_niveau_preuve_gradation.pdf)

**c) The final recommendations covered 11 topics from screening of high risk patients to palliative care. Chapter 7 of the Guidelines was devoted to surgical treatment (questions 36 to 45).**

**c1. Surgery : task-force subgroup.**

J.R.D ; delperojr@ipc.unicancer.fr

A.S. ; alain.sauvanet@aphp.fr‬‬‬‬

Emmanuel Buc MD PHD ;

Stéphanie Truant MD PHD ;

Lilian Schwarz MD PHD ;

c**2. Surgery : key questions (Q)**:

Q 36: How to manage the patient before surgery and what is the best timing for surgery ?

Q 37: What are the contraindications to resection related to the general status ?

Q 38: What are the criteria to define a standard PD for PDAC ?

Q 39: What are the criteria to define a standard DP for PDAC ?

**Q 40: What are the indications of vascular resection during pancreatic surgery for PDAC ? (J.R.D, A.S.) .**

Q 41: What are the indications of TP and/or extended visceral resections ?

Q 42: How to deal with intraoperative findings of liver metastasis, peritoneal carcinomatosis, paraaortic lymph node involvement ?

Q 43: Is there any indication for scheduled pallative surgery ?

Q 44: What are the minimum items that must be included in the operative protocol  ?

Q 45: What are the functional consequences after pancreatic surgery for PDAC ? how to manage these functional consequences ?

c**3**. **Literature search (with specific key words for Q 40*) :**

| Abstracts | 278 |
| --- | --- |
| *Exclusion* | *114* |
| Manuscripts | 164 |
| *Exclusion* | *72* |
| *First bibliography* | 92 |
| Result of Updated search until 6-2019 | 57 |
| Final bibliography | 149 |

Exclusion criteria :

• off topic ; not responding to the question

• Neuroendocrine tumors, solid pseudopapillary tumors, IPMN, benign tumors and low grade malignancies, other cancers;

• studies with few patients (<20), case reports, letters, editorials.

*** Q 40 : 150 references**

c**4**. **INCa coordinating team, guideline leader and working group**

**• INCa coordinating team** :

Gabriel FERRAND ([gferrand@institutcancer.fr](mailto:gferrand@institutcancer.fr));

Marianne DUPERRAY ([mduperray@institutcancer.fr](mailto:mduperray@institutcancer.fr));

Camille DE PERETTI ([cdeperetti@institutcancer.fr](mailto:cdeperetti@institutcancer.fr));

Tristan ROUE ([troue@institutcancer.fr](mailto:troue@institutcancer.fr));

Christine VERMEL ([cvermel@institutcancer.fr](mailto:cvermel@institutcancer.fr)).

**• Guidelines leader :**

Alain SAUVANET (ACHBT).

**• Working group (scientific society member):**

Jean Baptiste BACHET (FFCD), Erwan BORIES (SFED), Emmanuel BUC (SFCD), Christophe CASSINOTO (SIAD-SFR), Valerie CROISE-LAURENT (SIAD-SFR), Jerome CROS (SFP), Laetitia DAHAN (FFCD), Christèle DE LA FOUCHARDIERE (SNFGE), Jean-Robert DELPERO (ACHBT), Fadila FARSI (AFSOS), Stephane GARCIA (SFP), Florence HUGUET (FFCD), Stephane KOCH (SFED), Alain LUCIANI (SIAD- SFR), Vinciane REBOURS (SNFGE), Lilian SCHWARZ (SFCD), Jean-Pierre TASU (SIAD-SFR), Stéphanie TRUANT (ACHBT), Véronique VENDRELY (FFCD), Marie-Pierre VULLIERME (SIAD-SFR), Mathilde WAGNER (SIAD-SFR), Myriam WARTSKY ( Sociéte Francaise de Médecine Nucléaire).

All scientific societies were co-sponsors of the project.

c**4**. **External review**

The reviewers were identified by the regional cancer networks and 17 scientific societies (all specialties involved in the management of pancreatic cancer ; surgeons : 31%). Recommendations were sent by email to 126 reviewers (8th March 2019 - 8th - April 2019) with an online questionnaire (SPHINX); 70 complete responses were avalaible. Feedback from the national review was discussed at the last meeting of the working group on 9th May 2019.

*Q 40 : Agreement rate for the recommendations by the external reviewers*

*• do you agree with these recommendations?*

*
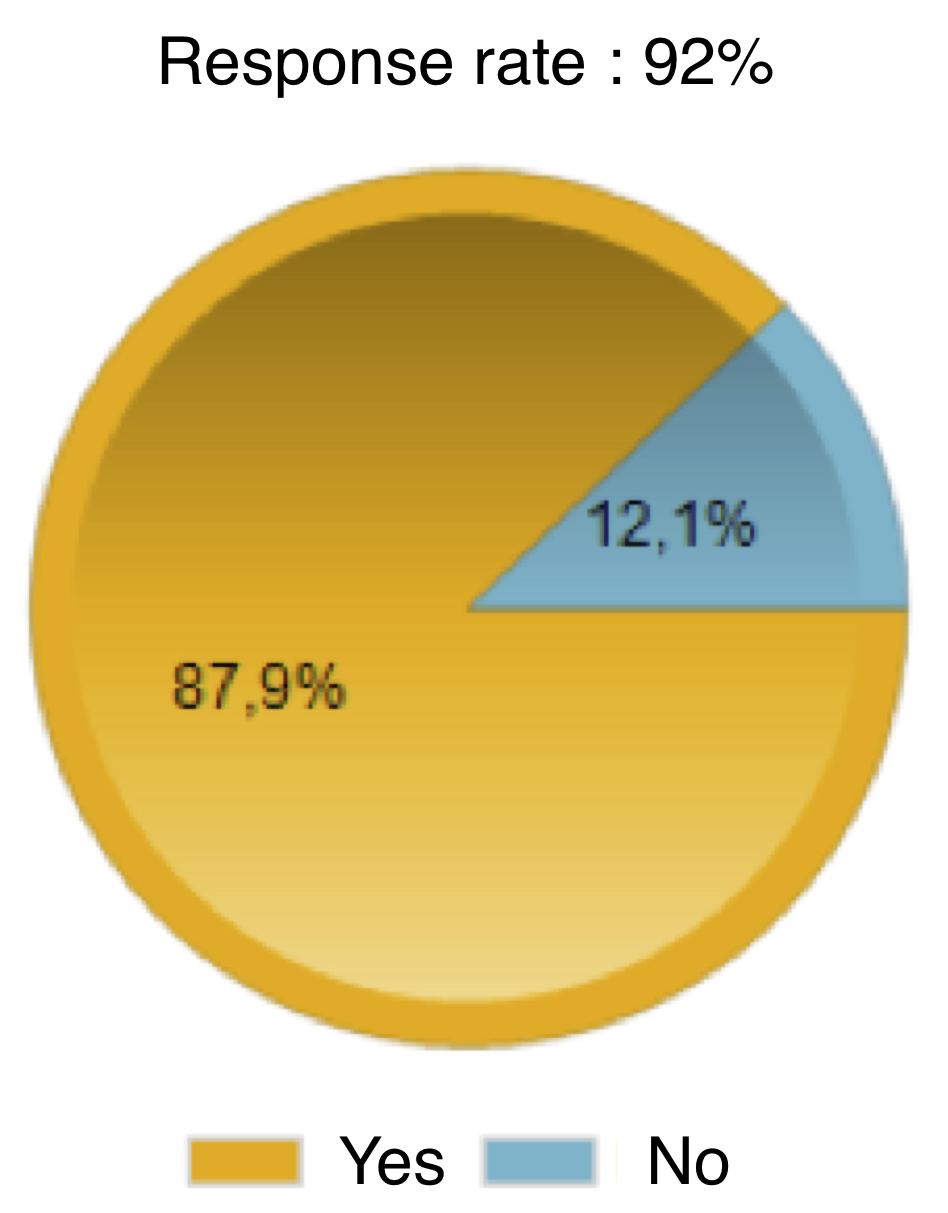
*

*
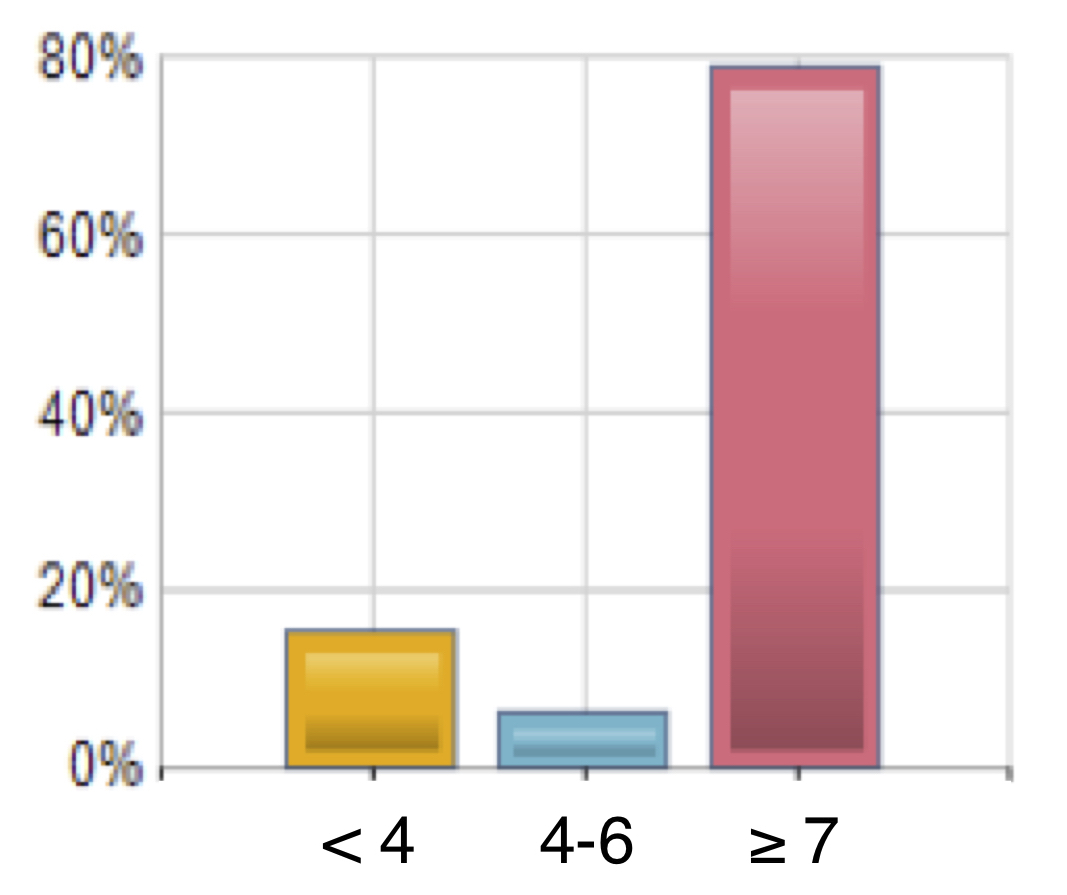
*

• 1 - 3 : Low agreement

• 4 – 6 : Moderate agreement

• 7 - 9 : Strong agreement

*• do you think these recommendations are applicable in your practice?*

*
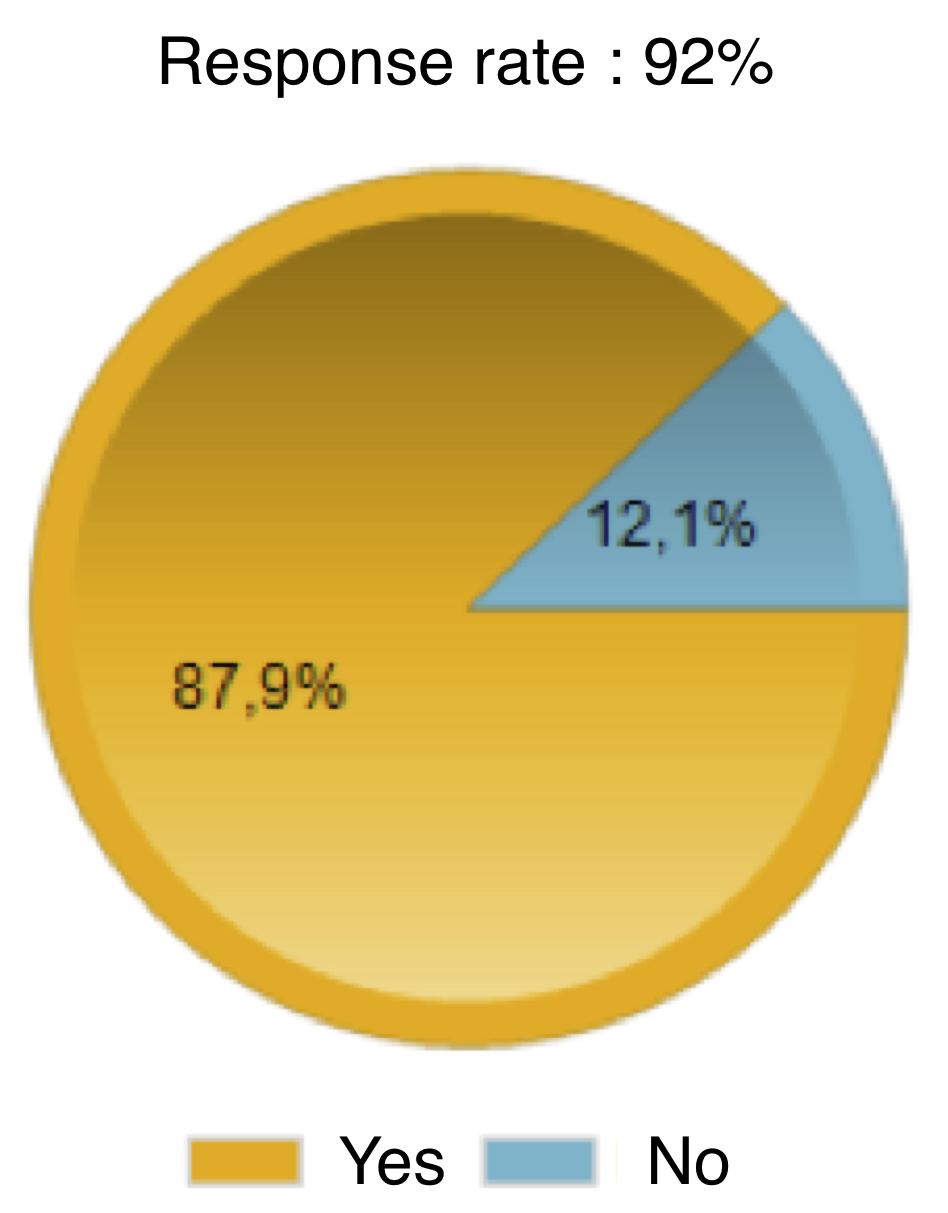
*

*
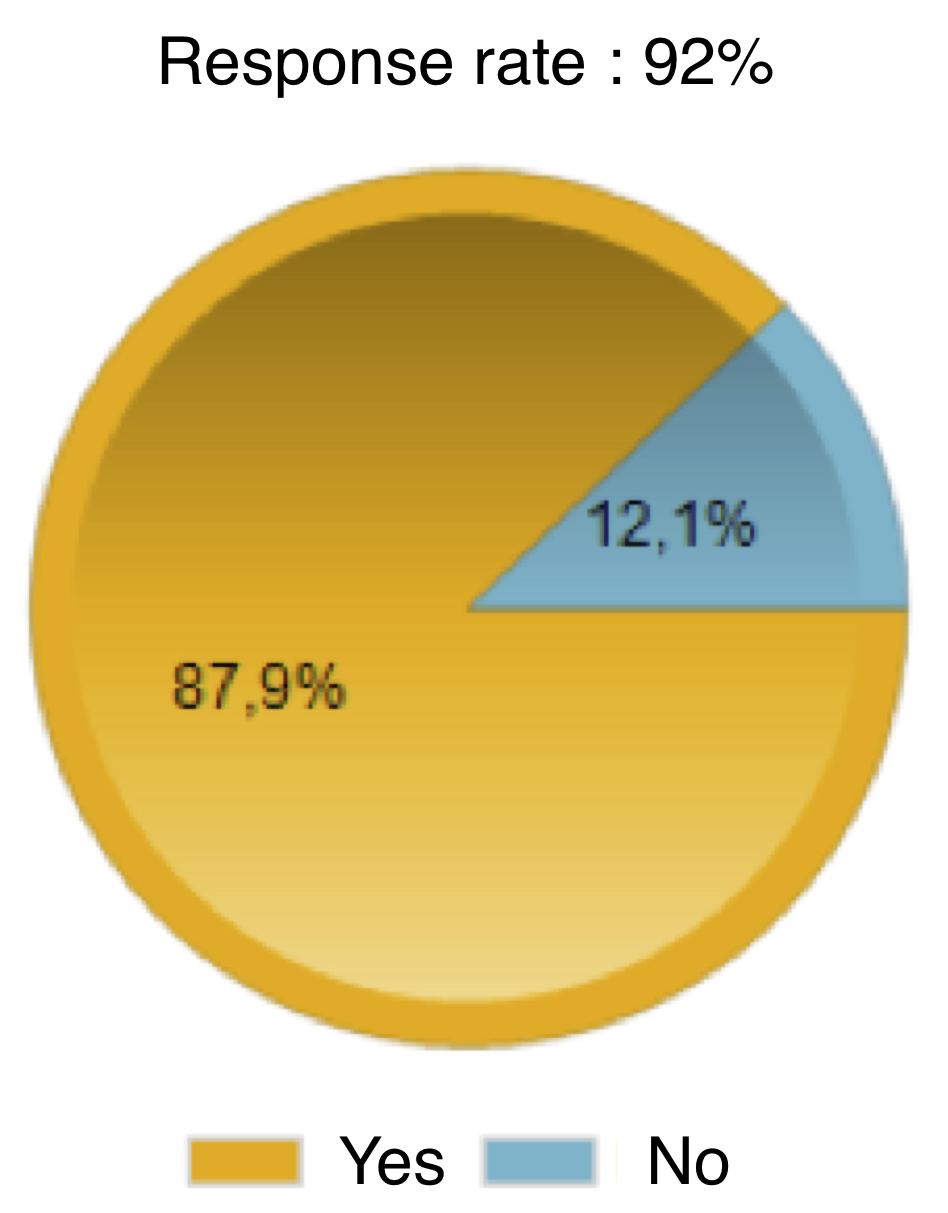
*

c**5**. **The current guidelines were co-sponsored by the following French scientific societies :**

• SIAD (Société d’imagerie abdominale et digestive): Society of abdominal and digestive imaging

• SFR (Société française de radiologie), French Society of Radiology ;

• SNFGE (Société Nationale Française de Gastroentérologie), French Society of Gastroenterology ;

• SFED (Société Française d’Endoscopie Digestive), French Society of Digestive Endoscopy

• FFCD (Fédération Française de Cancérologie Digestive) French Federation of Digestive Cancerology ;

• SFCD (Société Française de Chirurgie Digestive), French Society of Digestive Surgery ;

• SFMN (Société française de médecine nucléaire), French Society of Nuclear Medicine ;

• SFP (Société Française de Pathologie), French Society of Pathology

• AFSOS (Association Francophone pour les Soins Oncologiques de Support), French Association for Oncological Supportive Care.

c**6**. These Guidelines were funded by UNICANCER (section : National Program of Guidelines)

Label of the French National Cancer Institute (INCa) by decision of its President : N ° 2019-49, dated 02/09/2019, published in the « Official Bulletin for Health Protection and Solidarity ».
