# Supplementary material for: Vascular Resection for Pancreatic Cancer: 2019 French Recommendations Based on a Literature Review From 2008 to 6-2019
Source: Front Oncol. 2020 Feb 4;10:40. doi: 10.3389/fonc.2020.00040 (PMC7010716; doi:10.3389/fonc.2020.00040)

**Supplemental material 1.**

**NCCN** [**Pancreatic Adenocarcinoma, Version 1.2019.**](https://www.ncbi.nlm.nih.gov/pubmed/30865919)


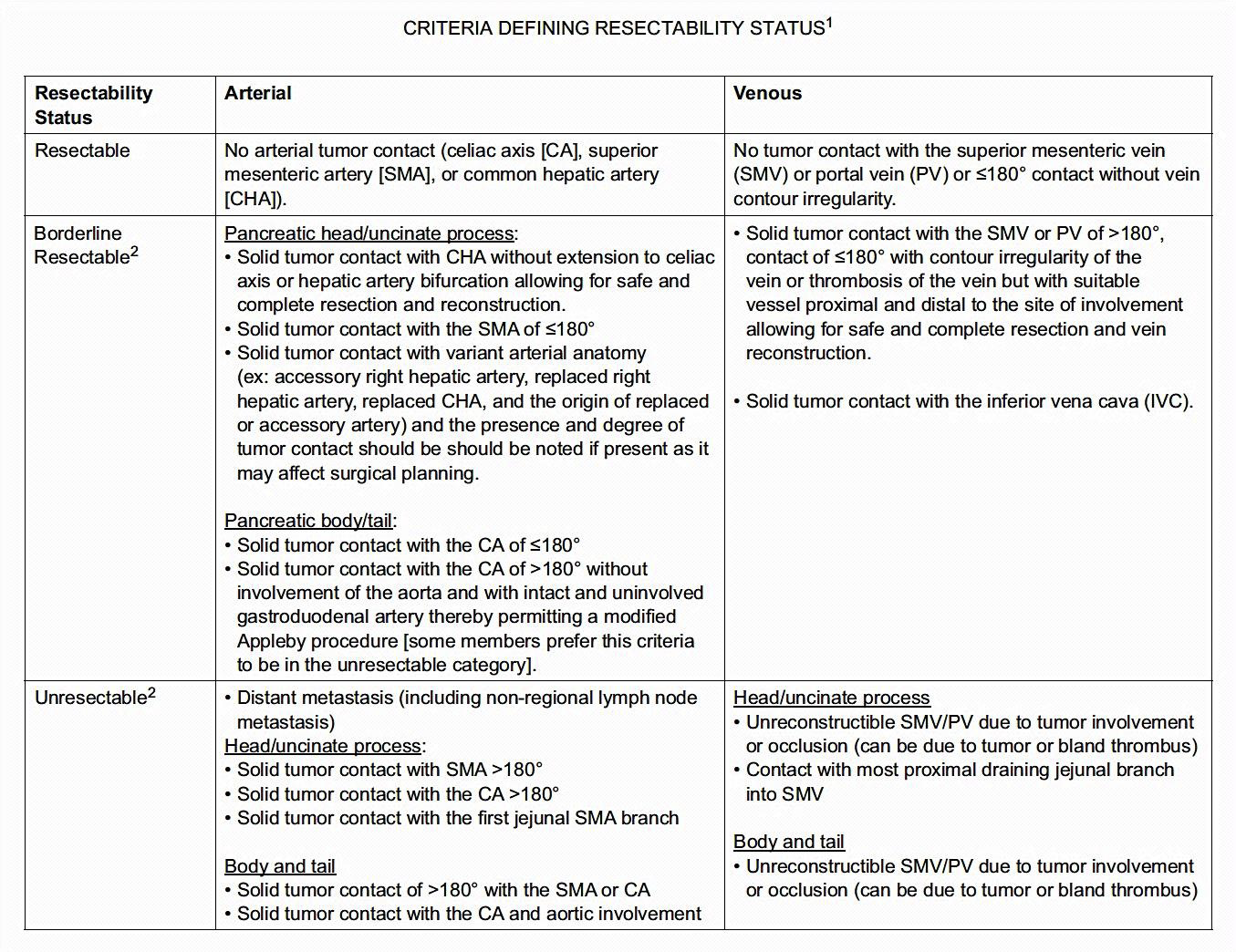


1• Al-Hawary MM, Francis IR, Chari ST, et coll. Pancreatic ductal adenocarcinoma radiology reporting template: consensus statement of the Society of Abdominal Radiology and the American Pancreatic Association. Radiology 2014 ; 270 : 248-260.

2• Solid tumor contact may be replaced with increased hazy density/stranding of the fat surrounding the peri-pancreatic vessels (typically seen following neo-adjuvant therapy); this finding should be reported on the staging and follow-up scans. Decision on resectability status should be made in these patients, in consensus at multidisciplinary meetings/discussions.

*“No randomized phase III trials have compared the approach of neo-adjuvant therapy in borderline resectable disease versus surgery without initial therapy, and the best regimens to use in the borderline neo-adjuvant setting are unknown. Although no high-level evidence supports its use, most NCCN Member Institutions now prefer an initial approach for patients with borderline resectable disease that involves neo-adjuvant therapy as opposed to immediate surgery. Several trials have shown that preoperative treatment of borderline resectable pancreatic adenocarcinoma can be effective and well-tolerated. For selected patients who appear technically resectable but have poor prognostic features (eg, markedly elevated CA 19-9 levels, large primary tumors, large regional lymph nodes, excessive weight loss, extreme pain), neo-adjuvant therapy can be considered after biopsy confirmation.”*

**Supplemental material 2.**

**ASCO recommendations. Potentially Curable Pancreatic Cancer: American Society of Clinical Oncology Clinical Practice Guideline Update.**

**In : Khorana AA et al. J Clin Oncol. 2017 ; 35 : 2324-2328**

**Recommendation 2.1: Primary surgical resection** of the primary tumor and regional lymph nodes is recommended for patients who meet all of the following criteria :

- no clinical evidence for metastatic disease,

- **performance status and comorbidity** profile appropriate for a major abdominal operation,

- **no radiographic interface** between primary tumor and mesenteric vasculature on high-definition cross-sectional imaging,

- a **CA 19-9 level** (in absence of jaundice) suggestive of potentially curable disease

(Type: evidence based, benefits outweigh harms; Evidence quality: intermediate; Strength of recommendation : strong).

**Supplemental material 3.**

**International consensus on definition and criteria of borderline resectable pancreatic ductal adenocarcinoma 2017. In : ISAJI S et al ; Pancreatology 18 (2018) 2-11**

**Defined patients with BR-PDAC according to the three distinct dimensions: anatomical (A), biological (B), and conditional (C).**

1. Anatomic factors include tumor contact with the superior mesenteric artery and/or celiac artery of less than 180° without showing stenosis or deformity, tumor contact with the common hepatic artery without showing tumor contact with the proper hepatic artery and/or celiac artery, and tumor contact with the superior mesenteric vein and/or portal vein including bilateral narrowing or occlusion without extending beyond the inferior border of the duodenum.

2. Biological factors include potentially resectable disease based on anatomic criteria but with clinical findings suspicious for (but unproven) distant metastases or regional lymph nodes metastases diagnosed by biopsy or positron emission tomography-computed tomography. This also includes a serum carbohydrate antigen (CA) 19-9 level more than 500 units/ml.

3. Conditional factors include the patients with potentially resectable disease based on anatomic and biologic criteria and with Eastern Cooperative Oncology Group (ECOG) performance status of 2 or more.

The definition of BR-PDAC requires one or more positive dimensions (e.g. A, B, C, AB, AC, BC or ABC).

**The present definition acknowledges that resectability is not just about the anatomic relationship between the tumor and vessels, but that biological and conditional dimensions are also important.**


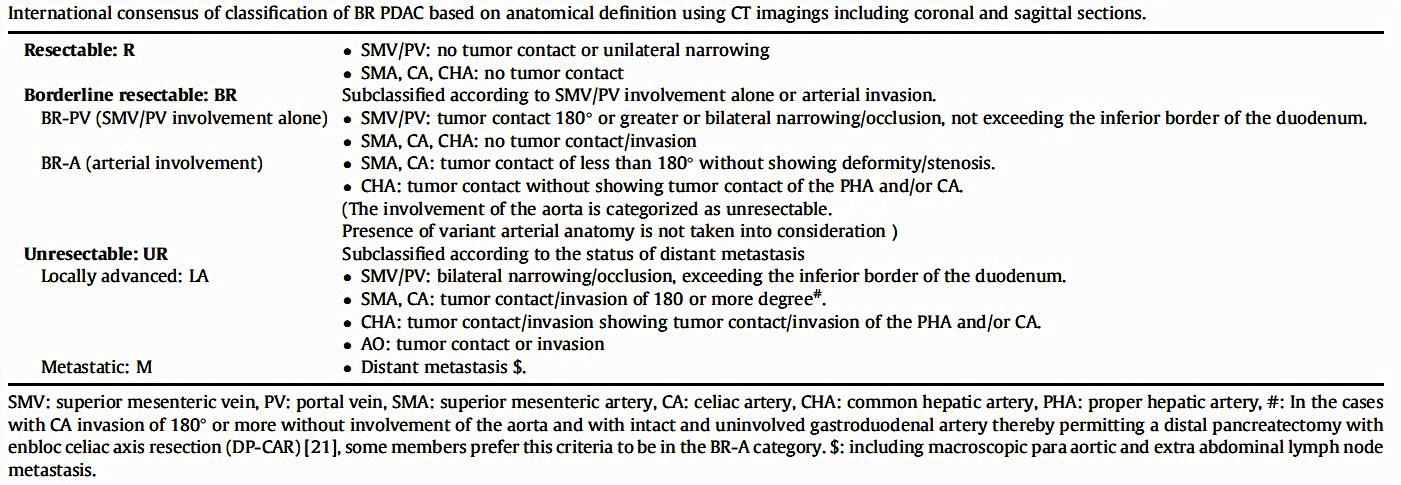


**Supplemental material 4.**

**Schorn S et al. The impact of neoadjuvant therapy on the histopathological features of pancreatic ductal adenocarcinoma - A systematic review and meta-analysis. Cancer Treat Rev. 2017 ; 55 : 96-106.**

**Relative Risks (RR) after neoadjuvant treatment (*versus* observed after upfront resection)**

| **Pathology** | **RR** | **95%CI** | **p** |
| --- | --- | --- | --- |
| \| **T1/T2** \| \| --- \| | \| 2.87 \| \| --- \| | 1.52- 5.42 | 0.001 |
| **T3/T4** | \| 0.78 \| \| --- \| | 0.69-0.89 | 0.0002 |
| **N0** | \| 2.14 \| \| --- \| | 1.85–2.46 | 0.00001 |
| **N+** | \| 0.59 \| \| --- \| | 0.53–0.65 | < 0.00001 |
| **R0** | \| 1.13 \| \| --- \| | 1.08–1.18 | < 0.00001 |
| **R1** | 0.66 | 0.58–0.76 | < 0.00001 |
| **Perineural inv.** | \| 0.78 \| \| --- \| | 0.73–0.83 | < 0.00001 |
| **Ly-vx-inv** | 0.50 | 0.36–0.70 | < 0.0001 |
| **Grade 3** | \| 0.82 \| \| --- \| | 0.71–0.94 | 0.005 |

**Supplemental material 5.**

Comprehensive Complication Index

# The CCI (CCI^®^-Calculator) can be readily computed on the basis of tabulated complications according to the Clavien-Dindo classification ; The final formula yields a continuous scale to rank the severity of any combination of complications from 0 to 100 in a single patient. (available at [www.assessurgery.com](http://www.assessurgery.com)).

# [Slankamenac K](https://www.ncbi.nlm.nih.gov/pubmed/?term=Slankamenac%20K%5BAuthor%5D&cauthor=true&cauthor_uid=23728278)^1^, [Graf R](https://www.ncbi.nlm.nih.gov/pubmed/?term=Graf%20R%5BAuthor%5D&cauthor=true&cauthor_uid=23728278), [Barkun J](https://www.ncbi.nlm.nih.gov/pubmed/?term=Barkun%20J%5BAuthor%5D&cauthor=true&cauthor_uid=23728278), [Puhan MA](https://www.ncbi.nlm.nih.gov/pubmed/?term=Puhan%20MA%5BAuthor%5D&cauthor=true&cauthor_uid=23728278), [Clavien PA](https://www.ncbi.nlm.nih.gov/pubmed/?term=Clavien%20PA%5BAuthor%5D&cauthor=true&cauthor_uid=23728278). The comprehensive complication index: a novel continuous scale to measure surgical morbidity. [Ann Surg.](https://www.ncbi.nlm.nih.gov/pubmed/23728278) 2013, 258 : 1-7.

#
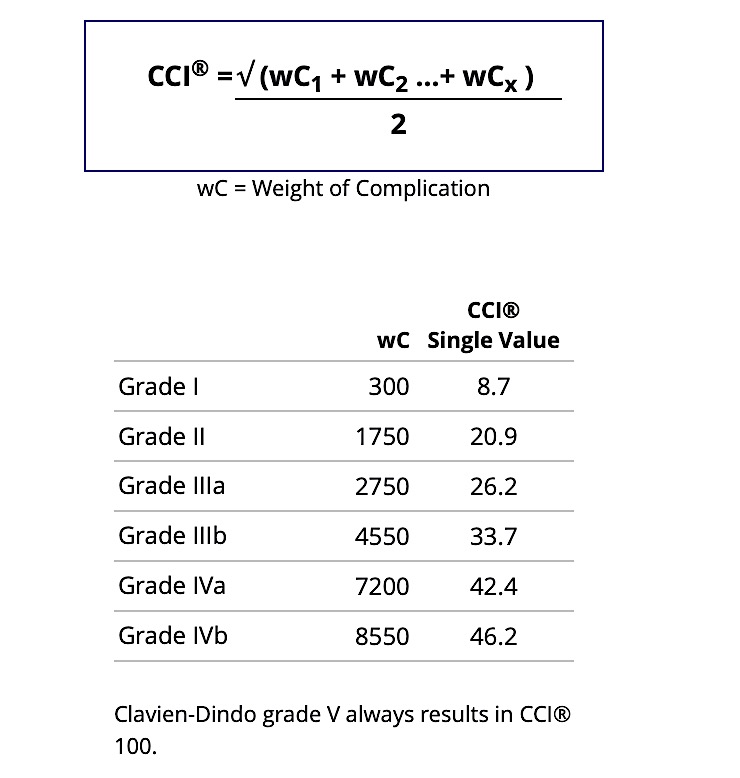

Supplement: Supplementary file 2 [file Data_Sheet_2.docx]
